# *In vivo* silencing of the thalamic Ca_V_3.1 voltage-gated calcium channels demonstrates their region-specific role in anesthetic mediated hypnosis

**DOI:** 10.3389/ebm.2025.10553

**Published:** 2025-05-16

**Authors:** Tamara Timic Stamenic, Simon Feseha, Brier Fine-Raquet, Vasilije P. Tadic, Slobodan M. Todorovic

**Affiliations:** ^1^ Department of Anesthesiology, University of Colorado, Aurora, CO, United States; ^2^ Department of Neuroscience, University of Colorado, Aurora, CO, United States; ^3^ Department of Pharmacology Graduate Programs, University of Colorado, Aurora, CO, United States

**Keywords:** calcium ion channels, thalamus, anesthesia, isoflurane, hypnosis

## Abstract

Although substantial progress has been made in the last three decades towards our understanding of how general anesthetics (GAs) act at the molecular level, much less is known about how GAs cause loss of consciousness at the level of neuronal networks. The role of thalamus as an important brain region in anesthetic-induced hypnosis is relatively well established, but the specific roles of voltage-gated ion channels in different functional regions of the thalamus in anesthetic mechanisms are not well studied. To address this gap in knowledge, we selectively silenced the *Cacna1g* gene that encodes the low-threshold-activated Ca_V_3.1 T-type voltage-gated calcium channel subunit by injecting short-hairpin RNA (shRNA) into midline and intralaminar - nonspecific thalamus (MIT) and sensory - specific ventrobasal (VB) thalamic nuclei in wild-type (WT) mice. Control animals were injected with scrambled shRNA. To validate our silencing approach, we performed patch-clamp experiments in acute thalamic slices *ex vivo*. In injected animals we determined anesthetic endpoints such as hypnosis measured with loss of righting reflex (LORR) and immobilization measured with loss of withdrawal reflex (LOWR) *in vivo* after administration of a traditional volatile GA isoflurane. Effective Ca_V_3.1 channel knock-down was documented by greatly diminished amplitudes of T-currents and absence of rebound burst firing in our patch-clamp recordings from thalamic slices. We found that knocking down Ca_V_3.1 channels in MIT significantly decreased inhaled isoflurane concentration that is required to induce LORR, but it did not affect speed of anesthetic induction and the immobilizing effect of isoflurane. In contrast, knocking down the Ca_V_3.1 channel in the VB thalamus did not affect any of the measured anesthetic endpoints. Hence, we concluded that Ca_V_3.1 channels in nonspecific MIT thalamus have a preferential role in anesthetic hypnosis when compared to the sensory VB thalamus.

## Impact Statement

General anesthetics (GAs) have been clinically used for nearly two centuries, but the mechanisms whereby different classes of these agents achieve different clinical effects are still not well understood. We found that knocking down CaV3.1 channels in MIT significantly decreased inhaled isoflurane concentration that is required to induce LORR, but it did not affect speed of anesthetic induction and immobilizing effect of isoflurane. In contrast, knocking down CaV3.1 channel in the VB thalamus did not affect any of the measured anesthetic endpoints.

## Introduction

General anesthetics (GAs) have been clinically used for nearly two centuries, but the mechanisms whereby different classes of these agents achieve different clinical effects are still not well understood. A complete anesthetic state involves loss of consciousness (hypnosis) and movement (immobilization), as well as loss of both pain sensation (analgesia) and recollection of the event (amnesia). Research advances in the last three decades strongly suggest that GAs act through specific sites on the neuronal membrane and that different ion channels that control neuronal excitability may mediate their clinical effects [1, 2]. It is well known that most GAs currently in use have either N-methyl-D-aspartate (NMDA) receptor-blocking or/and γ-aminobutyric acid A (GABA_A_) receptor-mimetic properties that can account for anesthetic hypnosis [1]. However, a family of neuronal voltage-gated calcium channels (VGCCs) was also implicated in the mechanisms of anesthesia because VGCC inhibition may be important in anesthetic action by decreasing neuronal excitability and presynaptic excitatory transmission [3].

Thalamus is one of the brain regions implicated in regulation of arousal, natural sleep-wake cycle and a very relevant site of anesthetic actions. The thalamus has traditionally been divided into three anatomical and functional groups: the principal (relay, sensory or motor) nuclei, the association nuclei, and the midline and intralaminar nuclei (MIT) [4, 5]. Since the sensory nuclei receive sensory information through ascending pathways and transmit it to distinct regions of the cortex, they are known as a specific part of thalamus [4, 5]. On the other hand, MIT are historically known as a nonspecific thalamus because of diffuse projections to different cortical and subcortical areas [4, 5]. Various thalamic nuclei are important in awareness, cognitive functions, and as targets for many GAs [1, 6, 7]. Importantly, most thalamic nuclei express different isoforms of T-type VGCCs that activate with small membrane depolarizations. The T-channels are crucial for the rhythmic oscillations between mutually interconnected cortical, inhibitory GABAergic neurons in the nucleus reticularis thalami (nRT) and glutamatergic relay neurons in the sensory thalamic nuclei (VB) and MIT. Our previous studies established that nRT neurons are enriched in Ca_V_3.2 and Ca_V_3.3 isoforms of T-channels that underlie their low-threshold-calcium spikes (LTSs) and burst firing [8]. In contrast to nRT, neurons of the VB and MIT express almost exclusively the Ca_V_3.1 isoform of T-channels [9–11]. In addition, we reported that Ca_V_2.3 R-type of VGCCs is also expressed in central medial nucleus of the thalamus (CMT, part of intralaminar thalamus) and in concert with the Ca_V_3.1 T-type channels regulate neuronal excitability [11, 12].

Recent findings have suggested that the intralaminar parts of thalamus (CMT) may act as a key hub through which GA-induced hypnosis and natural sleep are initiated [6]. Additionally, the CMT has been identified as the neuroanatomical site mediating the arousal response by manipulating activity of voltage-gated potassium channels [13–15]. Moreover, studies demonstrated that the paraventricular thalamus (PVT, part of the midline thalamus), is a key wakefulness-controlling nucleus in the thalamus [16, 17]. However, despite the proposed roles of the CMT and PVT in regulation of the state of arousal, the ability of GAs to regulate activity of VGCCs and specifically Ca_V_3.1 channels in MIT in the context of anesthetic endpoints is not well established.

We previously reported that T-currents in the CMT, nRT and VB are inhibited by clinically relevant concentrations of volatile GAs [11, 18, 19]. This strongly suggests that thalamic T-currents participate in anesthetic action, however, new genetic tools are needed for proof-of-concept studies for the role of T-channels in the clinical effects of GAs. Further, potential region-specific differences of T-channel inhibition within the thalamus are not known. To address this gap in knowledge, here we selectively silenced the *Cacna1g* gene that encodes the Ca_V_3.1 T-channel subunit by injecting short-hairpin RNA (shRNA) [20] into MIT and VB thalamic nuclei in wild-type (WT) mice. We then determined anesthetic endpoints such as hypnosis and immobilization after administration of a traditional volatile GA isoflurane.

## Materials and methods

**FIGURE 1:**
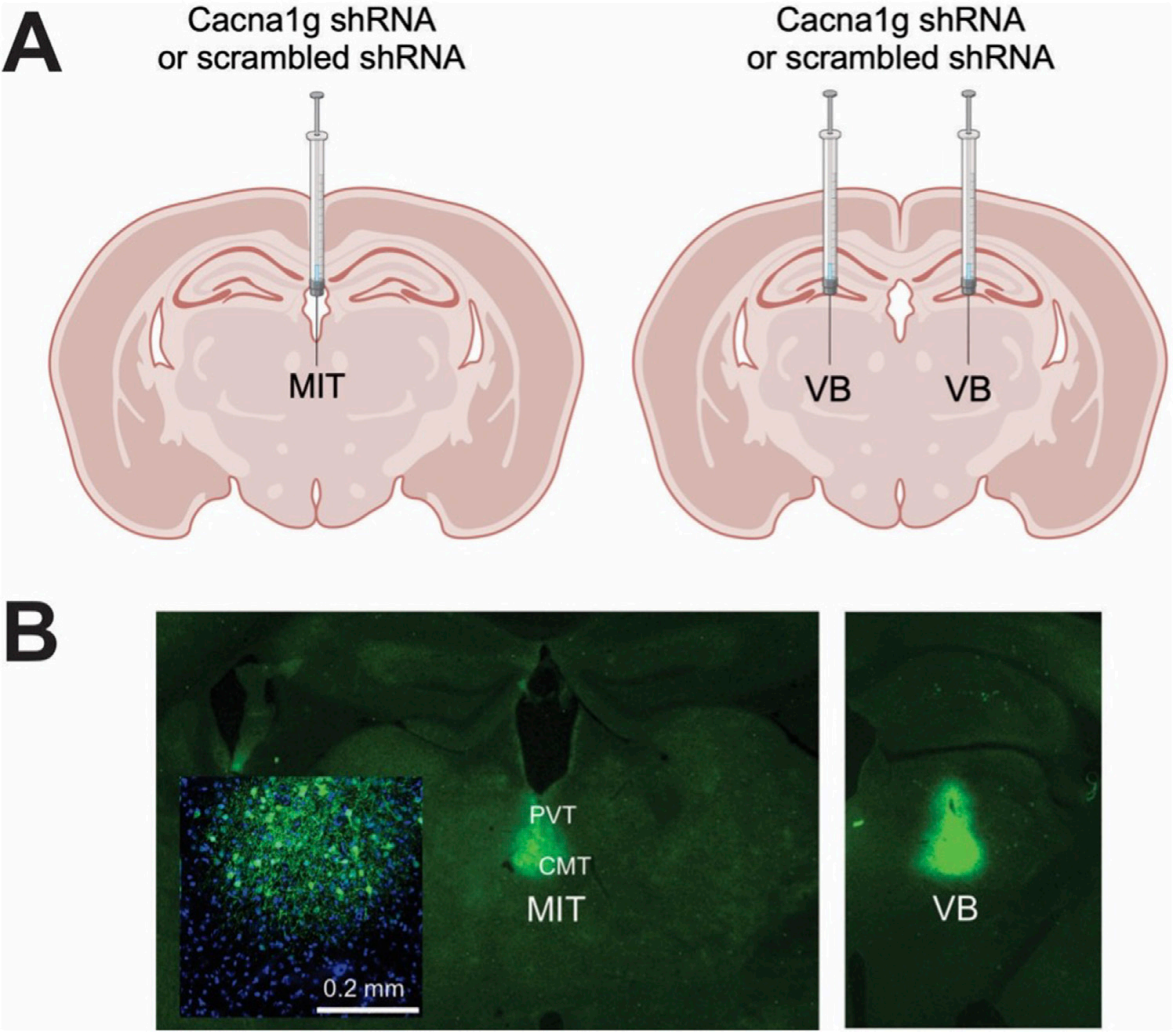
Generation of selective thalamic Ca_V_3.1 T-channels knock-down mice. **(A)** Diagram of stereotaxic injections; control (scrambled shRNA) or Cacna1g shRNA injection in the non-specific thalamus (midline and intralaminar thalamus - MIT) or specific – sensory thalamus (ventrobasal thalamus - VB). **(B)** Stereotaxic injections confirmation by immunohistochemistry.

To investigate the potential role of thalamic T-channels in anesthesia, we used a methodology which silences the Cacna1g gene that encodes the Ca_V_3.1 channel pore-forming subunit by injecting short-hairpin RNA (shRNA); this is the same procedure employed by others [21] and in our recent study [20]. In short, high-titer AAV2 vectors expressing Ca_V_3.1shRNA (*AAV2-GFP-U6-mCACNA1G-shRNA*) or control (scrambled shRNA) were obtained from Vector BioLabs, prepared and delivered either into the MIT targeting the intralaminar (CMT) and midline parts of thalamus (PVT) (in mm from bregma, AP: −1.35, ML: 0, DV: 3.95) or targeting both the right and left VB thalamus (AP: −1.75, ML: ±1.60, DV: 4.00) of WT male mice by stereotaxic injections using a 5-μL Hamilton syringe at a rate of 0.1 μL/min. Figure 1 shows a schematic of these areas. The induction of anesthesia in mice was performed in an anesthesia chamber with 3% isoflurane, after which they were placed on a stereotaxic frame and kept anesthetized via nose cone that continuously delivered 2-3% isoflurane throughout the whole procedure. Mice were monitored for the changes in the respiratory rate and the concentration of isoflurane was adjusted accordingly. The frame was equipped with automated drilling, followed by ultraprecise injection (Robot Stereotaxic, Neurostar). After surgery mice were treated with the analgesic Banamine 2 days and allowed to recover if no major neurological deficits were noticed. The effects of Cacna1g shRNA or control (scrambled shRNA) were studied at least 2 weeks following injections to allow adequate time for virus spread in the targeted areas.

### Immunohistochemistry (IHC)

Following completion of behavioral experiments, brains of all mice injected with Cacna1g shRNA or scrambled shRNA were processed for localization verification. Mice were deeply anesthetized with 5% isoflurane and transcardially perfused with phosphate buffered saline (PBS pH 7.4, Life Technologies), followed by 4% paraformaldehyde in 0.1 M phosphate buffer, pH 7.4 (PFA). Whole brains were extracted and post-fixed in PFA for 24 h. Brains were rinsed in PBS, embedded in 3% agarose and brain sections (50 μm) were prepared on a microtome (Leica VT1200). Slices were rinsed three times in PBS, mounted on slides and an antigen retrieval process was performed by exposing slides to a boiling citric buffer solution (0.1 M, pH 6.0). Sections were permeabilized in 1% glycine in PBST for 15 min and rinsed in PBS for 5 min. Sections were then blocked with 5% normal donkey serum in PBST (0.1% Triton X-100 in PBS) for 30 min, and incubated with primary antibody (rabbit anti-GFP; 1:1000; A11122; Invitrogen) diluted in 1% normal donkey serum in PBST overnight at 4°C. Slices were rinsed 3 × 5 min in PBST followed by a 5-min rinse in PBS and then incubated for 2 h with secondary anti-rabbit antibody (anti-rabbit Alexa 488: 1:500, Invitrogen) at room temperature and washed 3 × 5 min in PBS. Sections were coverslipped using a fluorescent mounting medium containing DAPI (Vector laboratories) and images were taken using a confocal laser scanning microscope (Olympus FluoView FV1200) at ×20 magnification using image stitching to obtain the entire region of interest. Although, in our stereotaxic injections we targeted the CMT, we found using confocal microscopy that GFP immunofluorescence has spread into adjacent nuclei [PVT and/or interomediodorsal nucleus of thalamus (IMD), Figure 1] in most of our experiments. Hence, due to this technical limitation we refer to nonspecific thalamic injections as targeting MIT, not just CMT. Only the mice that had viral GFP expression localized to the MIT or VB were included in analysis.

### Brain slice preparation for patch-clamp electrophysiology experiments

Patch-clamp experiments using brain slices from the virus-injected mice began at least 2 weeks after injection. Animals were anesthetized briefly with isoflurane, decapitated, and their brains rapidly removed. Fresh horizontal brain slices, 250 μm-thick, were sectioned at 4°C in a pre-chilled solution containing (in mM): sucrose 260, D-glucose 10, NaHCO_3_ 26, NaH_2_PO_4_ 1.25, KCl 3, CaCl_2_ 2, MgCl_2_ 2, using a vibrating micro slicer (Leica VT 1200S). Brain slices were immediately incubated for 45 min in a solution containing (in mM): NaCl 124, D-glucose 10, NaHCO_3_ 26, NaH_2_PO_4_ 1.25, KCl 4, CaCl_2_ 2, MgCl_2_ 2 at 37°C prior to use in electrophysiology experiments, which were conducted at room temperature. During incubation, slices were constantly perfused with a gas mixture of 95% O_2_ and 5% CO_2_ (v/v).

### Patch-clamp electrophysiology recordings

The external solution for whole-cell voltage-clamp recordings consisted of (in mM): NaCl 125, D-glucose 25, NaHCO_3_ 25, NaH_2_PO_4_ 1.25, KCl 2.5, MgCl_2_ 1, and CaCl_2_ 2. This solution was equilibrated with a mixture of 95% O_2_ and 5% CO_2_ (v/v) for at least 30 min with a resulting pH of approximately 7.4. The internal solution for recording well isolated T-currents consisted of (in mM): tetramethyl ammonium (TMA)-OH 135, EGTA 10, MgCl_2_ 2, and HEPES 40, titrated to pH 7.2 with hydrofluoric acid (HF) [22].

The current-voltage (I-V) curves were generated by stepping from the holding potential (Vh) of −90 mV to depolarized test potentials (Vt) from −80 to −40 mV in 2.5 mV increments. The voltage dependencies of steady-state activation were described with single Boltzmann distributions of the following forms: Activation: G(V) = G_max_/ (1 + exp[−(V - V_50_)/ *k*]); *G*_max_ is the maximal conductance (calculated by dividing current amplitude by estimated reversal potential), *V*_50_ is the voltage at which half of the current is activated, and *k* represents the voltage dependence (slope) of the distribution. The T-currents from the inactivation protocol were recorded by using a standard double-pulse protocol with 3.6-s-long prepulses to variable voltages (from −120 to −60 mV in 5 mV increments) and test potentials to −50 mV.

For the whole-cell current-clamp recordings, the internal solution consisted of (in mM): potassium-D-gluconate 130, EGTA 5, NaCl 4, CaCl_2_ 0.5, HEPES 10, Mg ATP 2, Tris GTP 0.5, pH 7.2. Glass micropipettes (Sutter Instruments O.D. 1.5 mm) were pulled using a Sutter Instruments Model P-1000 and fabricated to maintain an initial resistance of 3–5 MΩ. GFP-expressing thalamic neurons were identified using the microscope with epifluorescence and IR-DIC optics. Intrinsic excitability of thalamic neurons was characterized by using a multi-step protocol which consisted of injecting a family of depolarizing (50–300 pA) current pulses of 400 ms duration in 25 pA increments followed by a series of hyperpolarizing currents of the same duration stepping from −50 to −250 pA in 25 pA increments. Subsequent action potential (AP) tonic and rebound firing frequencies (per pulse and per burst) and input resistances were determined. The membrane potential was measured at the beginning of each recording and was not corrected for the liquid junction potential, which was around 10 mV in our experiments. The membrane input resistance was calculated by dividing the end of steady-state hyperpolarizing voltage deflection by the injected current. Neuronal membrane responses were recorded using a Multiclamp 700B amplifier (Molecular Devices, CA, United States). Voltage current commands and digitization of the resulting voltages and currents were performed with Clampex 8.3 software (Molecular Devices), and voltage and current traces were analyzed using Clampfit 10.5 (Molecular Devices).

### Animals

Experimental procedures with animals were performed according to the guidelines approved by the Institutional Animal Care and Use Committee (IACUC) of the University of Colorado Anschutz Medical Campus. Treatments of animals adhered to guidelines set forth in the NIH Guide for the Care and Use of Laboratory Animals. Our study was approved by the ethics committee of the University of Colorado Anschutz Medical Campus. The adult male C57BL/6J wild type (WT) mice (between 2 and 4 months of age) were used for behavioral experiments. C57BL/6J mice were obtained from the Jackson laboratory (USA). We opted for male mice, as very little is known about sex differences in activity of volatile anesthetics in the thalamus. All animals were maintained on a 14/10 h light-dark cycle with food and water *ad libitum*. Anesthetic endpoints were measured as we previously reported [23] as follows.

### Loss of righting reflex (LORR) and loss or withdrawal reflex (LOWR)

LORR is assessed by placing the mouse on its back until animal loses righting reflex. The criterion for the LORR is failure of mouse to right within a 30-s period. For LOWR, an alligator clip covered with airway tubing was used on proximal 1/3 tail and LOWR was considered when there was no withdrawal for a minimum of 30-s. All mice were placed on a heating pad in a chamber equilibrated with 0.5% isoflurane. Isoflurane was then increased by 0.1% every 10 min until LORR and LOWR was obtained. Illustration of the experiments with LORR and LOWR determination is depicted on top panels of Figures 4A, 5A.

### Anesthetic induction

Induction time was assessed by measuring the time to LORR (TTLORR) at a constant inhaled concentration of 1.2% isoflurane. Mice were placed on the heating pad in anesthetic chamber that was set at 1.2% isoflurane after a 30-min wait. Successful induction was determined when a mouse failed to right within a 30-s period.

### Drugs

Isoflurane was purchased from McKesson (San Francisco, CA) and Banamine (Merck) was obtained from the University of Colorado Hospital pharmacy.

### Data analysis

In every experiment, we attempted to minimize the number of animals used. All animals with complete data set and physiological parameters were included in the study. Statistical analysis was performed using two-way repeated measure (RM) ANOVA as well as student unpaired and two-tailed *t*-test, where appropriate. We used *Sidaks’s multiple comparisons test* where interaction between factors after two-way RM ANOVA was significant. Significance was accepted with *p* values < 0.05. Statistical and graphical analysis was performed using GraphPad Prism 8.00 software (GraphPad Software, La Jolla, CA, United States) and Origin 2018 (OriginLab, Northampton, MA, United States).

## Results

**FIGURE 2:**
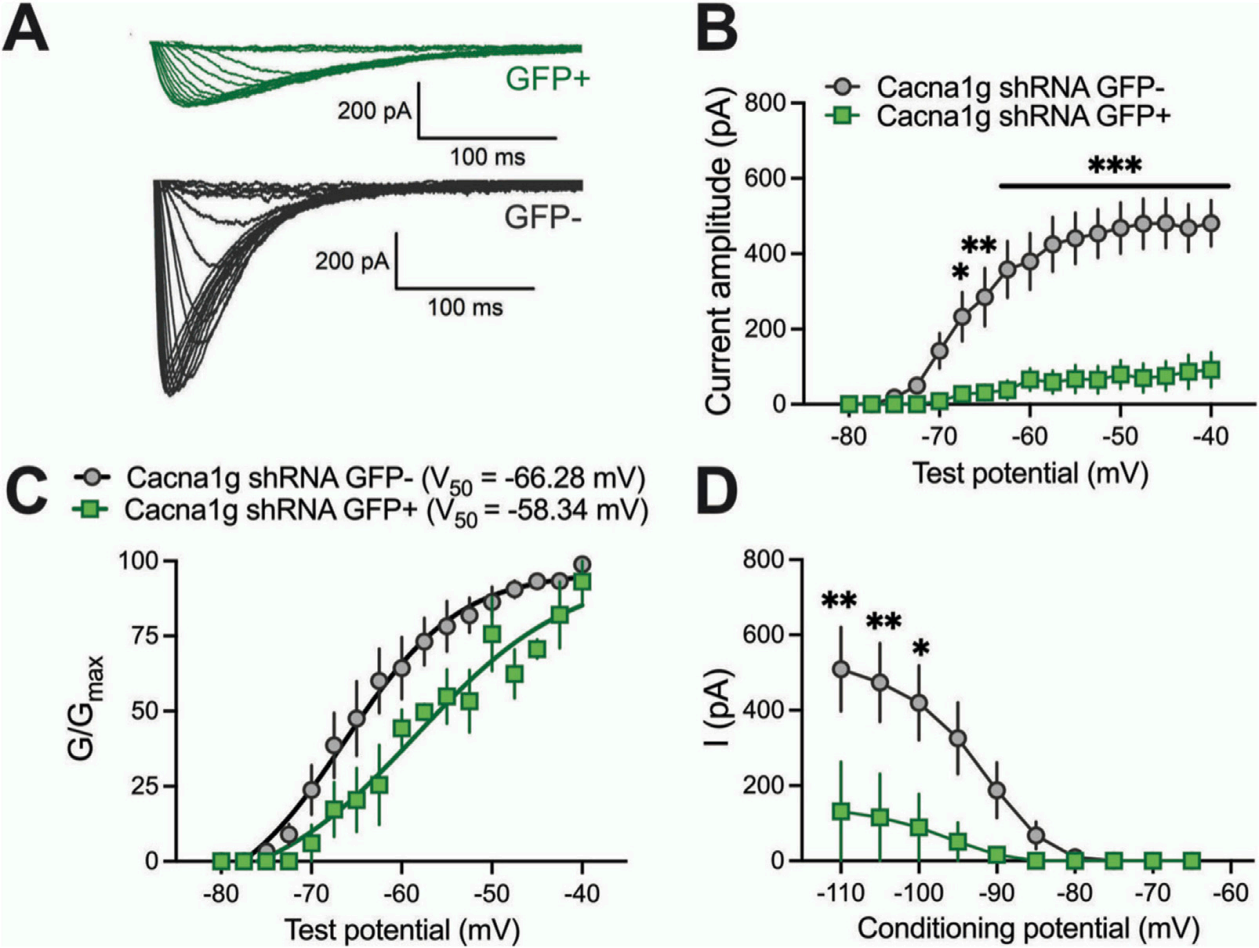
Biophysical properties of thalamic T-currents in Cacna1g shRNA GFP+ and GFP- neurons. **(A)** T-current *I*–*V* traces from representative GFP+ (green) and GFP- (black) neurons in the voltage range for *V*_t_ of −80 to −40 mV from an initial holding potential (*V*_h_) of −90 mV in 2.5 mV increments. Note that the T-currents recorded from the GFP+ cells are smaller and don’t show typical cris-crossing pattern. **(B)** Average T-current amplitude, as calculated from the steady-state activation protocol was reduced in Cacna1g shRNA GFP+ neurons in comparison to GFP- cells (two-way RM ANOVA: interaction F_(16,129)_ = 12.54, p < 0.001; test potential F_(16,192)_ = 25.01, p < 0.001, GFP F_(1,12)_ = 27.34, p < 0.001; Sidak’s *post hoc* presented on Figure). N = 7 cells per group, note that 3 GFP+ cells did not have T-currents. **(C)** The average voltage dependence of a steady-state activation (*G*/*G*_max_) curve with *V*_50_ value noted on the graph for GFP+ and GFP- thalamic neurons. The difference between *V*_50_ for T-current activation was not statistically significant between GFP+ and GFP- cells. N = 3 GFP+, N = 7 GFP-neurons. **(D)** Average T-current amplitude, as calculated from the steady-state inactivation protocol (double-pulse protocol with 3.6-s-long prepulses to variable voltages (from −120 to −50 mV in 5 mV increments) and a test potential (*V*_t_) of −50 mV) was greatly reduced in Cacna1g shRNA GFP+ neurons in comparison to GFP- cells (two-way RM ANOVA: interaction F_(9,72)=_4.61, p < 0.001; potential F_(9,72)_ = 12.24, p < 0.001, GFP F_(18)_ = 4.99, p = 0.056; Sidak’s *post hoc* presented on Figure). N = 4 GFP+, N = 6 GFP- cells per group, note that just one of four GFP+ cells had measurable T-currents. *p < 0.05, **p < 0.01, ***p < 0.001.

**FIGURE 3:**
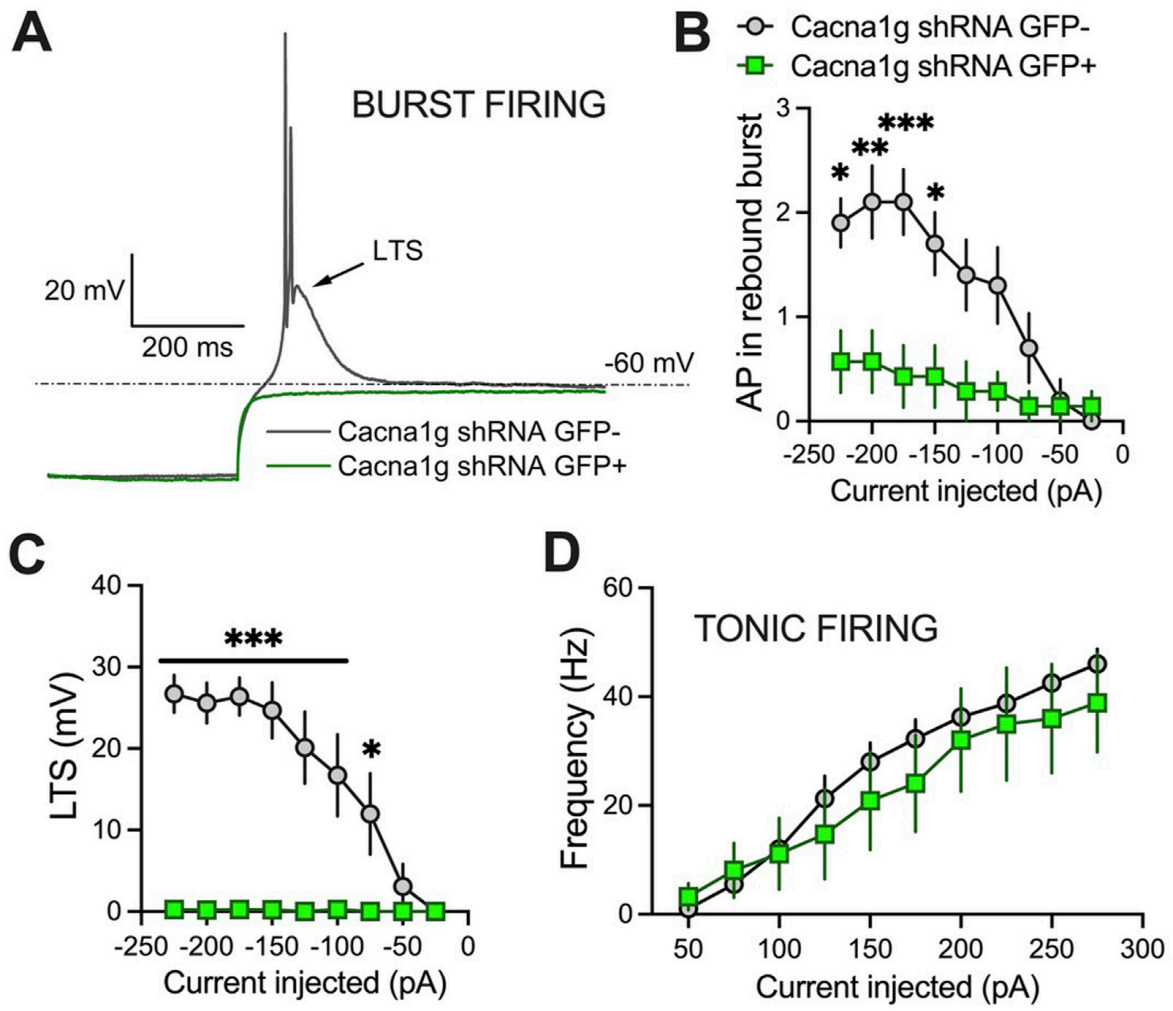
Excitability differences between GFP+ and GFP- thalamic neurons from Cacna1g shRNA injected animals. **(A)** Original traces from representative thalamic neurons recorded from GFP+ (green) and GFP- (gray) neurons show active membrane responses to a hyperpolarizing (−225 pA) current injection. Note that GFP+ neuron does not show APs nor low threshold spike (LTS) after membrane hyperpolarization. **(B)** Number of action potentials (AP) in rebound burst was statistically significant smaller in GFP+ neurons (two-way RM ANOVA: interaction F_(8,120)=_5.34, p < 0.001; current injected F_(8,120)_ = 11.95, p < 0.001, GFP F_(1,15)_ = 9.07, p = 0.009; Sidak’s *post hoc* presented on Figure). **(C)** LTS was not observed in GFP+ neurons (two-way RM ANOVA: interaction F_(8,120)=_12.06, p < 0.001; current injected F_(8,120)_ = 12.51, p < 0.001, GFP F_(1,15)_ = 31.07, p < 0.001; Sidak’s *post hoc* presented on Figure). **(D)** Graph of averages of tonic AP firing frequency and current injections of 50–275 pA from multiple experiments shows no difference between GFP+ and GFP- Cacna1g shRNA injected thalamic neurons. N = 7 GFP+, N = 10 GFP- cells. *p < 0.05, **p < 0.01, ***p < 0.001.

To validate functional knock-down of T-currents we first performed patch-clamp recordings using acute brain slices from WT mice injected with Cacna1g shRNA (Figure 2). Transfected neurons were readily identified in our recordings from live brain slices with bright GFP immunofluorescence. Specifically, we compared T-current biophysical properties from the acute thalamic slices in GFP positive (GFP+) and GFP negative (GFP-) neurons. T-currents were evoked using our standard current-voltage (I-V) protocols with depolarizing steps to test potentials (Vt) from −80 to −40 mV from holding (Vh) potentials of −90 mV. Original traces of inward calcium currents from representative recordings in the CMT in GFP+ neurons (green traces) and GFP- neurons (black traces) are depicted in Figure 2A. On average, we found that peak T-currents in GFP+ neurons (n = 7, green symbols) were almost completely abolished as evidenced by about 90% decreased amplitudes when compared to GFP- neurons (n = 7, gray symbols) across most of the test potentials in our I-V recordings (Figure 2B). We next compared steady-state activation curves in two groups and found that GFP+ neurons exhibited a small but insignificant depolarizing shift in V_50_ of channel activation when compared to GFP- neurons (Figure 2C). Finally, we used an independent protocol of recordings T-current amplitudes (Vt −50 mV) after conditioning pre-pulses from −110 to −60 mV. Average graphs from these experiments showing largely decreased T-current amplitudes in GFP+ neurons (green symbols) when compared to GFP- neurons (gray symbols) is summarized on Figure 2D. Our results showing greatly decreased T-current amplitudes strongly suggest that excitability of GFP+ neurons may be decreased as result of injections of Cacna1g shRNA. Hence, in ensuing current-clamp experiments we compared tonic and burst firing properties of GFP+ and GFP- neurons in CMT as summarized on Figure 3. Original traces of a rebound action potential (AP) and a T-channel-dependent low-threshold-calcium spike (LTS) from a GFP- neuron (gray trace) are depicted on Figure 3A. The same figure shows a lack of rebound APs and completely abolished LTS in a GFP+ neuron (green trace). In the summary graph on Figure 3B we depict the average number of rebound APs in GFP- neurons (n = 10 neurons, gray symbols) resulting from progressively stronger hyperpolarizing steps from −50 to −250 pA. In contrast, the same figure shows only minimal active membrane response upon injections of the same currents (n = 7 neurons, gray symbols). Likewise, Figure 3C shows that in the same two groups when we compared LTSs are completely absent in GFP+ neurons (green symbols) while in GFP- neurons (gray symbols) they show typical voltage-dependence with larger amplitudes associated with stronger current injections. As expected, we found very little difference in the two groups when we compared properties of tonic firing of APs in response to escalating depolarizing current injections from +50 to +300 pA (Figure 3D). Overall, our patch-clamp recordings from thalamic neurons *ex vivo* in animals injected with Ca_V_3.1shRNA show that GFP+ neurons exhibited largely decreased T-current amplitudes and greatly diminished rebound burst firing.

**FIGURE 4:**
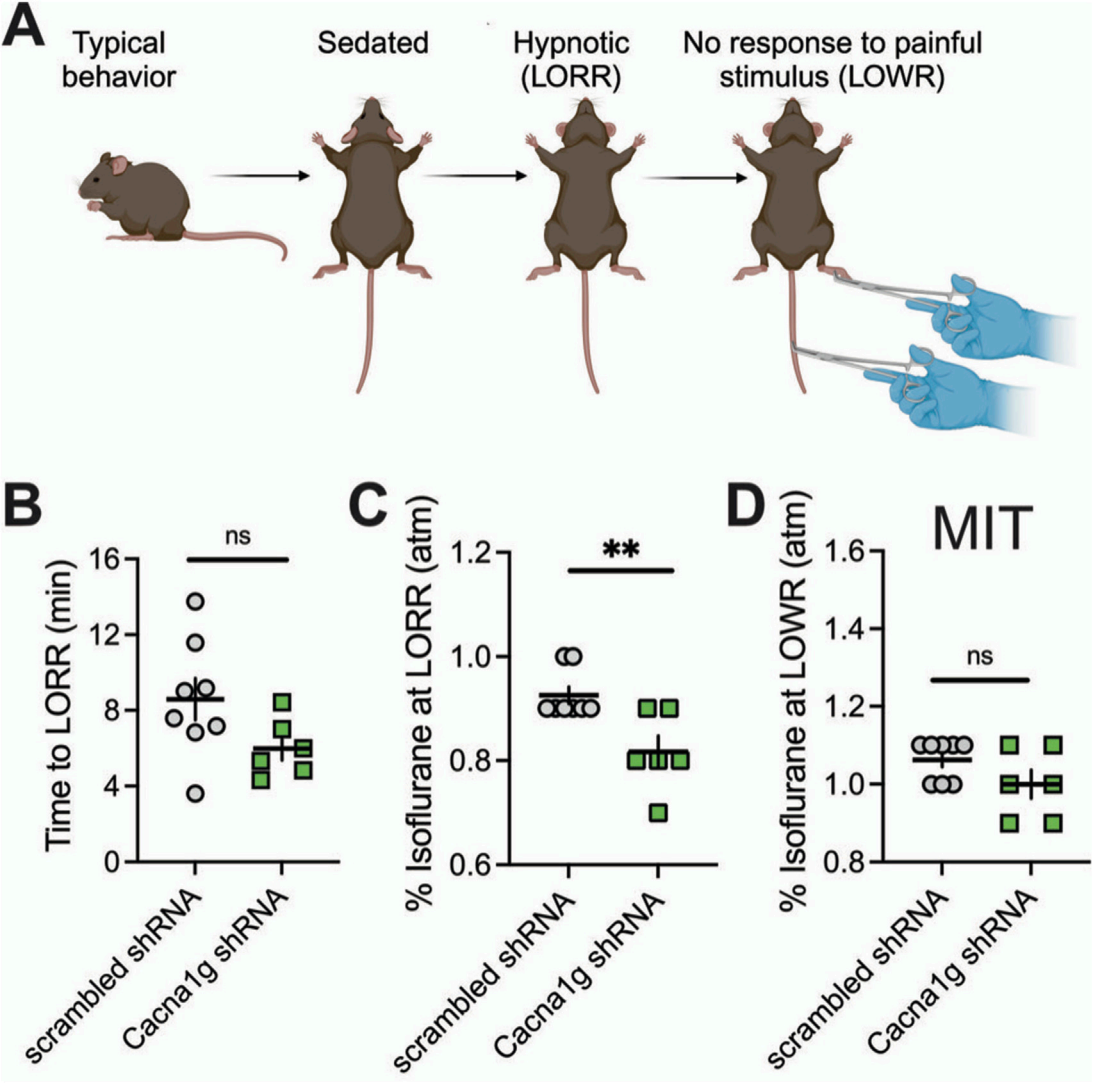
Effect of knocking-down of Ca_V_3.1 T-channels from MIT on LORR. **(A)** Schematic representation of LORR and LOWR experiments. **(B)** Time to LORR was not statistically significant between control (scrambled shRNA) and Cacna1g shRNA MIT injected mice. **(C)** Cacna1g shRNA MIT injected animals required less isoflurane for achieving LORR (hypnotic effect) in comparison to control male mice (unpaired two-tailed t-test: t_(12)_ = 3.34, p = 0.006). **(D)** There was no difference in isoflurane requirement for LOWR between control scrambled shRNA and Cacna1g shRNA MIT injected animals. N = 8 control scrambled shRNA and N = 6 Cacna1g shRNA animals. **p < 0.01.

**FIGURE 5:**
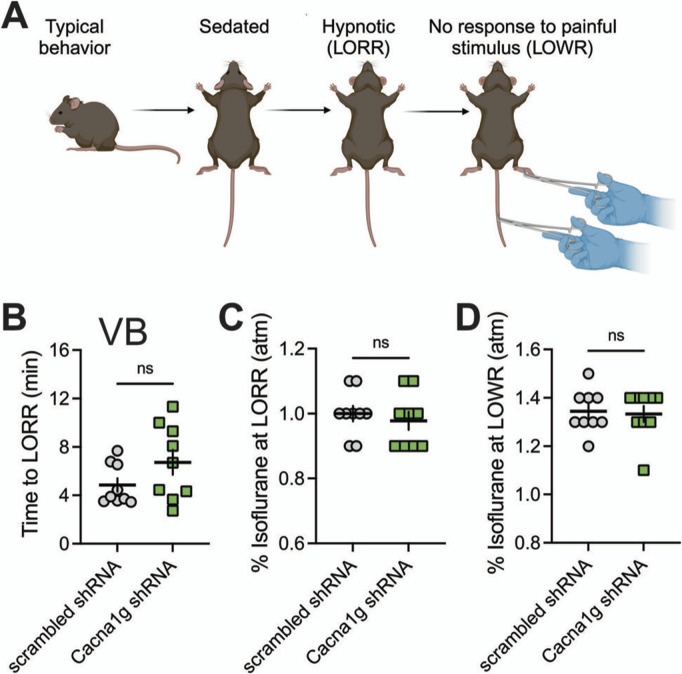
Effect of knocking-down of Ca_V_3.1 T-channels from VB on LORR. **(A)** Schematic representation of LORR and LOWR experiments. **(B)** Time to LORR was not statistically significant between control (scrambled shRNA) and Cacna1g shRNA in VB injected mice. **(C)** There was no difference in isoflurane requirement for LORR (hypnotic effect) between control and Cacna1g shRNA in VB infected mice. **(D)** There was no difference in isoflurane requirement for LOWR between control and Cacna1g shRNA in VB injected animals. N = 9 control scrambled shRNA and N = 9 Cacna1g shRNA animals.

In the ensuing *in vivo* experiments, we tested control groups (mice injected with scrambled shRNA) and experimental groups (mice injected with Cacna1g shRNA) for anesthetic endpoints, as determined by concentrations of inhaled isoflurane required to induce LORR. The examiner was blinded to the experimental and control groups. Figure 4 summarizes our data from the experiments where the Cacna1g shRNA or scrambled shRNA (control group) were injected into MIT. We found no significant difference in speed of induction as measured by TTLORR in Cacna1g shRNA group (n = 6, green symbols) when compared to our controls (n = 8, scrambled shRNA, gray symbols) as depicted on Figure 4B. In contrast, we found about 10% decrease in inhaled % atm isoflurane needed to induce LORR in group where Cacna1g shRNA was injected in the MIT region (Figure 4C). Finally, we found that the immobilizing effect measured by LOWR after injections of Cacna1g shRNA and scrambled shRNA into MIT region was not different in the two groups (Figure 4D). In contrast, mice injected with Cacna1g shRNA in the VB region (n = 9) did not show any difference in the speed of anesthetic induction (Figure 5B), or concentration of isoflurane required to induce LORR when compared to controls (n = 9) (Figure 5C). Similarly to injections to MIT, we found that LOWR response was not different between Cacna1g shRNA (n = 9) and scrambled shRNA (n = 9) group in our VB injections (Figure 5D). We conclude that *in vivo* silencing of Ca_V_3.1 channels in two functionally different regions of the thalamus, such as the MIT and the VB, differentially affected the required hypnotic concentration of isoflurane without an apparent difference in the speed of anesthetic induction and immobilizing properties of isoflurane.

In the ensuing *in vivo* experiments, we also tested the ability of mice injected either with scrambled shRNA or with Cacna1g shRNA into the MIT or the lateral thalamus targeting bilateral VB to perform behavioral tasks in an open-field test. We found that performance of mice during 10 min of testing in the groups injected either with scrambled or Cacna1g shRNA when compared to the naive, uninjected mice, was not different in the open-field testing (n = 5–10 mice per group, data not shown). Hence, it appears that intra-thalamic injection of viral vectors did not cause any major neurological deficit *per se* because it did not affect the general motor ability of mice.

## Discussion

One of the compelling reasons to study VGCCs in the mechanisms of anesthetic actions is that these channels are essential in regulation of synaptic transmission and excitability in the neuronal sleep pathway such as thalamus. The thalamus is the major gateway for the flow of sensory information from the periphery to the cortex and the disruption of thalamocortical connectivity may be an essential common feature of the hypnotic effects of many GAs. Indeed, both human and animal studies *in vivo* have indicated that the thalamus is deactivated during anesthesia [24]. Two thalamic regions are particularly relevant for our considerations as neuronal network targets for GAs. One such region, the thalamic VB nucleus, receives direct sensory projections from the periphery and projects mostly to the barrel cortex, it is a principal thalamic nucleus often referred to as a specific or sensory thalamus [5, 25]. The other region of interest, known as a nonspecific thalamus, consists of the CMT and PVT, parts of the MIT, which project diffusely to the different cortical and subcortical areas [5]. The parts of MIT are interposed between the brain stem “arousal” system and are ideally suited to control the overall level of thalamic and cortical activity [5, 26, 27]. Importantly, our previous *ex vivo* studies using acute brain slices have demonstrated that Ca_V_3.1 channel-mediated excitability in both the VB [19] and in the CMT [11] is diminished with clinically relevant concentrations of isoflurane. Hence, we hypothesized that knocking-down Ca_V_3.1 channels in the CMT and VB neurons may have different effects on loss of consciousness induced by GAs. To test this hypothesis, we produced region-specific knock-down of Ca_V_3.1 channels in MIT and VB nuclei to determine if diminished excitability of these neurons *ex vivo* correlate with the ability of a traditional volatile GA such as isoflurane to induce hypnosis in mice. These experiments addressed a long-standing unresolved issue of whether inhibition of Ca_V_3.1 T-channels in different functional regions of the thalamus is important for GA-induced hypnosis. Most thalamic neurons fire APs in a regular (tonic) mode when depolarized from the resting membrane potential, and high frequency burst firing mode that crowns low-threshold calcium spikes (LTS) if they are hyperpolarized sufficiently to de-inactivate T-type calcium channels [11, 28]. Indeed, we showed that knocking down Ca_V_3.1 channels in thalamus completely abolished LTS and greatly diminished rebound burst firing pattern while it had only a minimal effect on the tonic firing pattern (Figure 3). Our voltage- and current-clamp experiment validated our silencing technique using an shRNA approach that we also used to study the functional role of Ca_V_3.1 channel in the subiculum [20]. A previous study used a global knockout mouse to investigate the role of Ca_V_3.1 channels in anesthetic sensitivities. Petrenko and colleagues reported that global Ca_V_3.1 KO mice showed no change in anesthetic requirements (isoflurane, halothane, sevoflurane, pentobarbital) for LORR and LOWR but have delay in the onset of anesthetic induction measured by TTLORR [29]. They further concluded that the timely induction of anesthesia/hypnosis by volatile anesthetic agents and some intravenous anesthetic agents may require the normal functioning of the Ca_V_3.1 channel isoform. However, since Ca_V_3.1 channels are expressed in many different parts of the thalamus, hippocampus and cortex, we opted for the knock-down approach to be able to target specific thalamic regions and minimize compensatory changes in neuronal circuitry that are more likely with global knockouts. In contrast to this study with global KO mice, we found that knocking down this T-channel isoform in MIT leads to a decreased requirement for hypnosis induced by isoflurane. Our finding is consistent with another study that examined the specific role of the VB and CMT in anesthetic hypnosis [6]. Specifically, Baker and colleagues recorded local field potentials (LFPs) from four brain regions (barrel neocortex, VB, anterior cingulate cortex, and CMT) simultaneously in freely moving rodents during transitions into natural sleep and anesthetic-induced (propofol and dexmedetomidine) LORR. They found that for propofol-induced LORR and natural sleep, the LFP changes with neuronal oscillations occur first in the nonspecific thalamus before changes could be detected in the neocortex. With dexmedetomidine, they found that initial LFP changes occurred simultaneously in the nonspecific thalamus and neocortex. Overall, they concluded that CMT acts as key hub through which both anesthetic hypnosis and natural sleep are initiated. Our results are consistent with the idea that the nonspecific MIT region of the thalamus is more important for isoflurane-induced hypnosis than the specific sensory VB thalamus. In addition, we validated important role of Ca_V_3.1 isoform of T-type channels in this mechanism of anesthetic hypnosis. Towards this end, we recently reported that other classes of GAs like neuroactive steroids induce hypnosis in rodents at least in part by inhibiting Ca_V_3.1 channels in the thalamus [30, 31]. Hence, our future experiments will address the issue if Ca_V_3.1 T-type channels in MIT region of the thalamus show a similar preferential role in neurosteroid-induced hypnosis.

## Data Availability

The raw data supporting the conclusions of this article will be made available by the authors, without undue reservation.
